# Supplementary material for: Are carbon emissions trading and green financial instruments synergistic? -Comprehensive quantitative research based on content analysis
Source: PLoS One. 2024 Mar 7;19(3):e0298601. doi: 10.1371/journal.pone.0298601 (PMC10919601; doi:10.1371/journal.pone.0298601)
Supplement: S1 Data — (ZIP) [file pone.0298601.s001.zip › Data/地级市Z_F工作报告.zip!/║■▒▒/╢⌡╓▌╩╨/└┤╫╘╣1⁄2╓┌║┼í╛┬φ┐╦╩2╛▌═°í┐_2017.docx]

各位代表：

　　现在，我代表市人民政府向大会报告工作，请予审议，并请各位市政协委员和列席人员提出意见。

　　一、过去五年工作回顾

　　过去五年，是鄂州改革创新、克难攻坚、团结奋斗、砥砺前行的五年，是鄂州综合实力提升最快、城乡面貌变化最明显、生态文明建设力度最大、人民群众受益最多、城市影响力和竞争力大幅提升的时期。特别是2013年7月22日习近平总书记亲临鄂州视察指导工作，让我们倍感荣耀，深受鼓舞。在习近平总书记系列重要讲话特别是视察湖北、视察鄂州重要讲话精神指引下，在市委的坚强领导和市人大、市政协的监督支持下，我们凝神聚力，务实重行，积极推动结构调整，实现了经济平稳增长、社会全面发展。

　　过去五年，主要取得以下成绩：

　　生态保护彰显新作为。坚定不移把长江大保护摆在压倒性位置，把生态优先、绿色发展理念贯穿于发展全过程。梁子湖区被列为全国生态文明建设试点，500平方公里全面退出一般工业。主动退垸（湖、田）还湖，涂镇湖、曹家湖、垱网湖等一批湖泊重回母亲湖怀抱。推进长江岸线生态保护，关停高能耗、高污染企业500多家，取缔非法码头和砂场99个。开展环境空气质量改善攻坚战，PM10、PM2.5大幅下降。建成集镇污水处理厂和农村污水处理设施583座，在全省率先建成城乡一体垃圾全收集全处理系统。节能减排任务超额完成，万元GDP能耗持续降低。

　　转型发展实现新突破。正确处理稳中求进与结构调整之间的关系，积极培育新增长动能，促进经济发展换道超越。建成中部规模第一的电子商务基地，引进了唯品会、亚马逊、苏宁云商等电商龙头企业，社会消费品零售总额增速领跑全省。加强市校合作，引进华科大鄂州工研院等一批创新主体，成功转化全数字PET-CT等科技项目100多项，发明专利授权量年均增幅居全省前列。新兴产业年均增速超过24%，高新技术产业增加值占工业增加值比重达30.1%。企业主体加速成长壮大，工业规模企业净增235家，销售过亿元企业净增160家。设立8亿元投资引导基金，引进30亿元中经盛世创新投资基金。顾地科技成功上市，57家企业在“新三板”、“四板”挂牌。创建驰名商标7件、湖北著名商标和名牌产品达108个。建成生态农业基地413个，获得有机认证证书51张。地理标志证明商标总数居全省第二，“三品一标”农产品达到166个。农民专业合作社总数达1413家，家庭农场达708家。农业增加值增幅居全省前列。完成配电网改造资金10.5亿元，农村电力基础设施显著改善。

　　改革开放释放新动力。城乡空间规划、基础设施、产业布局、要素交换、公共服务和社会管理“六位一体”城乡一体化发展模式基本形成。深化行政管理体制改革，横向大部制，纵向扁平化，加大“放管服”，推进市级职权下放、公共服务下沉和“四张清单一张网”。全面取消非行政许可审批，市级行政审批事项减至221项。推进行政事业单位资产运营制度改革，昌达公司累计融资10.96亿元。推进农村产权制度改革，完成农村集体土地确权颁证，“五权”抵押融资贷款累计达3.47亿元，建设用地挂钩指标交易额累计达10.04亿元。全国中小城市综合改革、公立医院改革、“多规合一”试点、商事登记制度改革等多点突破。三江港港口码头工程加快建设，铁路货运枢纽项目正式动工。2016年4月，国家民航局正式批复湖北国际物流核心枢纽机场选址鄂州，在我市产业发展和城市建设历史上具有重要里程碑意义。

　　城乡建设呈现新面貌。八大新区竞相发展格局已经形成，基础设施建设和产业集聚步伐明显加快，建成发展大道、鄂东大道、凤凰大道、红莲大道等园区重大基础设施，工业园区面积达102.3平方公里，葛店开发区升级为国家级经济技术开发区。汉鄂高速、吴楚大道、武黄武冈城际铁路、鄂州大道南互通建成通车，鄂咸高速、主城区客运综合枢纽等一批重大交通项目加快推进。累计完成城建投资超过300亿元，建成市新博物馆、西山公园、橘园公园等重点市政工程。完成棚户区改造3.4万套、保障性住房1.2万套。“五城同创”取得阶段性成果，城市管理水平显著提高，美丽乡村建设彰显山水田园特色，城乡面貌焕然一新。

　　民生福祉得到新提升。五年累计完成民生投入超过260亿元，占财政支出的比重达75%。城乡居民人均收入稳步增长，差距缩小，农民收入基本实现翻番。发放各类救助资金6.1亿元，城市和农村低保标准分别提高到500元/月和350元/月。累计实现37207名贫困人口脱贫、22个贫困村出列。累计发放小额担保贷款3.6亿元，实现新增就业11.8万人。城乡医保实现“三网合一”，报销比例居全省前列。建成中心医院综合楼、市精神卫生中心、市实验小学、市第二幼儿园。华中师范大学梁子湖校区获教育部批准立项。鄂州职业大学进入国家骨干（示范）高职院校行列。文体场馆全部免费对市民开放，《梁子湖传说》等文艺精品创作成绩显著。在全省率先实现被征地农民社会保障全覆盖、率先实现城乡一体化供水、率先实现义务教育均衡发展、率先实现特困群众兜底保障、率先实现村村通客车。平安法治鄂州建设深入推进，“六五”普法圆满收官。安全生产隐患排查标准化、数字化体系建设走在全国前列。构建“1+N”社会矛盾纠纷预防化解体系，强化社会治安综合治理，人民安居乐业，社会和谐稳定。

　　外事、统计、审计、保密、方志、档案、气象、地震、新闻出版广电、民族宗教、人民防空、食品药品、防灾减灾、对外援助、妇女儿童、老龄、残疾人、公共资源交易、社会科学等各方面工作都取得了新的进步。

　　过去五年，全市地区生产总值（GDP）年均增长9.7%；规模以上工业增加值年均增长11.3%；地方公共财政预算收入年均增长14.6%；固定资产投资年均增长24.3%；社会消费品零售总额年均增长13.2%；城镇登记失业率控制在4%以内。主要指标在合理区间运行，基本完成七届人大一次会议确定的预期目标，实现了经济社会持续平稳健康发展。人均GDP、工业化率、城镇化率等指标居全省前列。城市软实力不断提升，荣获全国旅游标准化示范城市、全国绿化模范城市、省“双拥”模范城、省级文明城市、“宽带中国”示范城市等荣誉称号。

　　五年来，我们把全面从严治党要求贯穿政府工作各方面，高度重视政府自身建设。自觉接受市人大及其常委会的法律监督和工作监督，主动接受市政协的民主监督，共办理市人大代表建议378件、市政协提案1394件。认真贯彻中央八项规定精神，完成公车改革，“三公”经费大幅下降，“四风”整治和廉洁政府建设成效显著。

　　刚刚过去的2016年，稳增长调结构任务繁重，保稳定保平安压力巨大，经济发展逆水行舟，社会建设负重前行。面对复杂严峻的形势，我们应急谋远，多措并举，砥砺奋进，经济社会发展缓中趋稳、稳中向好，主要指标保持较快增长。预计全年地区生产总值增长8%，规模以上工业增加值增长8.5%，地方公共财政预算收入增长10.5%，固定资产投资增长17%，社会消费品零售总额增长13.4%，外贸出口增长15.9%，城镇居民收入增长8%，农村居民收入增长8%，实现了“十三五”良好开局。

　　产业结构调整步伐加快，高新技术产业占GDP比重提高到16.4%，第三产业提高到32%；引进一批创新平台和55个科技创新团队，81个“市校合作”项目深入推进。新登记市场主体增长41.7%，增幅居全省第一。坚持科学防汛、生态防汛，战胜98+特大洪涝灾害，对14处圩垸实施永久性退垸还湖，面积达8.71万亩。新签约投资亿元以上项目58个，签约总金额929亿元。省级重点项目完成投资72亿元，市级重点项目完成投资308亿元。供给侧结构性改革成效明显，压减钢铁产能222万吨，关停2台36平方米烧结机，金融机构杠杆率更趋合理，开展直供电交易改革，通过降税降价降费降低企业成本6.4亿元以上，商品房去库存周期由上年的14个月下降到9.4个月。开展自然资源资产负债表编制和领导干部自然资源资产离任审计试点。开工市政项目113个，加快推进市民中心、就业中心、凤凰大桥、东洋澜湖生态修复、重载车专用通道、老城区雨污分流改造等重点工程建设。全国卫生城市创建通过国家暗访检查，全国文明城市创建强力推进。14个贫困村出列、9999名贫困人口脱贫，社会大局保持平安稳定。

　　各位代表，五年的成绩得益于市委正确领导，得益于全市人民团结拼搏，得益于社会各界大力支持。特别是去年入汛以来，面对历史罕见的洪涝灾害，全市上下万众一心，最大努力保卫人民群众生命财产安全，最大力度推进灾后重建。在抗击重大自然灾害的关键时刻，共产党人的时代风范和社会各届众志成城、保卫家园的团结互济精神在大灾之年得到充分体现，这是我们推动事业持续发展的宝贵精神财富和强大动力。

　　各位代表，过去的成绩来之不易，我代表市人民政府，向各位人大代表、政协委员，向各民主党派、工商联、无党派人士、人民团体，向人民解放军、武警官兵，向所有参与、支持、关心鄂州建设发展的同志们、朋友们，表示衷心的感谢和崇高的敬意！

　　同时我们也必须清醒地认识到，个别工作未能实现预期目标；结构调整阵痛、动能转换困难、经济增速换挡相互交织；城市规划建设管理水平不高，生态环境脆弱，良好的交通区位和生态优势还没有转化为发展优势；改革试点面临深层次体制机制障碍；社会事业发展滞后，人力资源结构不优，城市文明程度和法制建设水平有待提升，城市治理能力和治理体系与建设国际航空大都市的要求还有差距；“12·2”重大道路交通安全事故教训深刻。对此，我们不回避，不推诿，不懈怠，振奋精神，全力以赴，认真加以解决。

　　二、未来五年的目标任务

　　各位代表，今天的鄂州，处于全新的发展方位，面临转型发展的历史性拐点，机遇与挑战并存，机遇大于挑战。随着长江大保护战略的实施，梁子湖在长江流域中的战略性功能更加突出，生态价值、社会价值和经济价值日益凸显。随着武鄂黄黄一体化特别是武鄂同城化深入推进，基础设施共建，公共服务共享，产业协同发展，生态环境共治，我市的区位优势正加速转变为生产要素和产业集聚优势，尤其是科教和人力资源的导入有利于弥补发展短板、注入持续动力。更为重要的是，湖北国际物流核心枢纽机场落户鄂州，必将极大提升我市在全国内陆开放格局中的战略地位，使我们可以在更大范围和更高层次上整合国内国外两个市场、两种资源，成为强力支撑我市跨越发展的重大战略平台。

　　各位代表，诸多战略机遇叠加，我们从此站在一个新的发展起点上，站在了鄂州“换道超越”的历史“风口”，从来没有像今天这样如此接近城市现代化目标。在刚刚闭幕的市第七次党代会上，市委明确指出从“钢城”向“港城”发展的战略方向，高瞻远瞩地描绘了建设国际航空大都市的宏伟蓝图，令人鼓舞，催人奋进。今后五年，我们将按照市委的战略部署，以敢为人先、时不我待的担当和气魄，全力加快建设国际航空大都市。

　　今后五年工作的总体要求是：高举中国特色社会主义伟大旗帜，以马克思列宁主义、毛泽东思想、邓小平理论、“三个代表”重要思想、科学发展观为指导，全面贯彻党的十八大和十八届三中、四中、五中、六中全会精神，深入贯彻习近平总书记系列重要讲话精神，紧紧围绕“五位一体”总体布局和“四个全面”战略布局，以第一力度抓第一要务，自觉践行新发展理念，适应把握引领经济发展新常态，以提高发展质量和效益为中心，以推进供给侧结构性改革为主线，坚持产业政策要准、创新政策要活、改革政策要实、社会政策要托底的政策思路，全面做好稳增长、促改革、调结构、惠民生、防风险各项工作，不断创造鄂州速度、质量、效率、标准，全面建成更高标准小康社会，开启城市现代化建设新征程，加快建设国际航空大都市，为湖北“建成支点、走在前列”作贡献、打头阵！

　　今后五年的主要目标是实现“四个提升”：

　　——综合经济实力提升。实现地区生产总值1300亿元，年均增长9%，人均GDP超过10万元，居全省前列，经济总量在全省位次前移。固定资产投资年均增长15%。经济结构明显优化，服务业占GDP比重达40%以上。

　　——人民幸福指数提升。脱贫攻坚任务如期完成，公共服务体系更加健全，城镇登记失业率控制在4%以内，城乡居民收入较快增长、差距缩小，教育现代化和健康鄂州建设取得重大进展，社保体系更加完善，文体事业和文化产业共同发展。资源利用、环境质量、生态保护、绿色生活、公众满意度等绿色发展指标优于国家考核标准。

　　——城市治理能力提升。城市管理体制机制逐步完善，管理机构优化整合，管理和执法能力进一步加强，平安法治鄂州建设水平显著提升，系统完备、科学规范、运行有效的城市治理体系基本形成，精细化管理水平显著提高。

　　——依法行政能力提升。政府机构、职能、权限、程序、责任法定化水平进一步提高，依法决策机制更加健全，行政执法体制更加完善，政务公开全面推进，行政执法责任制全面落实。

　　在工作着力点上，要争做“五个示范”：

　　——争做绿色发展示范，探索在保护中更好发展、在发展中更加有效保护的发展新模式。将生态环境保护与培育新兴产业、改善人民群众生活环境有机结合起来，实现环境、经济、社会协调发展。大力实施山水田林湖生态保护和修复工程，全面提升环境保护标准。统筹空间规划、用途管制、产业政策、开发强度、生态补偿、绩效考核、综合执法等全过程管控，提升生态治理能力。探索反映市场供求稀缺程度的自然资源价格发现机制，创新生态治理模式，推动市域范围内横向生态补偿，积极为国家生态文明建设提供先行先试制度示范。

　　——争做科技创新示范，走出新经济引领、科技创新驱动的发展新路径。以现代临空产业为突破口，强力引进前沿性、引领性的科技龙头企业和创新资源，形成大协作、大配套、大集群，做大做强创新底盘。深化供给侧结构性改革，以创新推动传统产业技术改造和优化升级。进一步发挥梁子湖自然生态优势和城市圈科教技术资源优势，加大对创新平台和创新主体培育力度，全面优化创新环境，实现创新企业、创新要素、创新平台、创新环境良性互动。整合政策引导、基础设施、公共服务、社会配套等各种资源，为创新人才营造良好的干事创业环境。

　　——争做深度开放示范，搭建多式联运、联通国内外市场的开放新平台。全力建设湖北国际物流核心枢纽机场，确保“2020年大飞机在鄂州起飞”。加快建设三江港铁路枢纽和综合码头，推动与全省主要开放口岸连通建设，发展铁、水、公、空多式联运综合交通体系，为国际国内货物集散提供基础设施支撑。积极争取有利于国际空港和临空经济区开发开放的国家支持政策，充分发挥机场对提升城市功能和聚集产业的战略作用，最大限度地分享开放成果。加快推动武鄂黄黄一体化发展，在更大范围内促进人力资源和产业资源合理流动、高效配置。

　　——争做综合改革示范，打造以制度创新释放动力、空间、效益的发展新引擎。发展出题目，改革做文章。深化改革的主攻方向是为国际航空大都市建设提供动力支持和政策保障，着力提高城市治理能力，完善治理体系。适应现代化城市内在发展要求，进一步整合政府职能，提升行政效能。完善政府和社会资本合作模式（PPP），不断创新管理方式，进一步加大对社会资本开放力度。继续深化行政、经济、社会管理、科技、文化、生态文明、社会事业等领域改革，坚决破除一切不利于生产力发展的体制机制障碍。

　　——争做新型城镇化示范，建设以人为本、精细管理、精明增长的江南美丽田园城市。围绕国际航空大都市建设，按照城市、产业、生态相辅相成、相互促进的要求，统筹空间、规模、产业三大结构，统筹规划、建设、管理三大环节，统筹改革、科技、文化三大动力，逐步实现生产、生活、生态协同发展。落实总体发展战略，统筹协调各类规划，形成城乡统筹、协调发展的格局。开展城市设计和修补，传承城市精神和历史文脉，城乡建设要望得见山，看得见水，记得住乡愁。按照“强功能、补短板、提品质”的要求，加快城市建设步伐，切实提升城市管理标准和管理水平。创新城市治理方式，提高市民文明素质，实现城市协同善治。

　　三、2017年重点工作

　　今年全市经济社会发展的主要预期目标是：地区生产总值增长9%左右，规模以上工业增加值增长8.5%，固定资产投资增长15%左右，地方公共财政预算收入增长10%左右，社会消费品零售总额增长13%左右，外贸出口增长10%，城乡居民收入与经济增长同步，城镇新增就业1.8万人。完成省定节能减排目标。

　　重点抓好以下七个方面工作：

　　（一）强力推进空港核心区建设

　　全面启动机场建设。积极推进可研报告编制和报批，推进总体规划、机场初步设计获得国家相关部门批复。以湖北国际物流核心枢纽机场项目和葛店中部电商基地为基础，申报设立海关特殊监管区域。全面开展机场核心区征地拆迁工作，加快燕矶、杨叶2个还建安置小区建设。

　　突破发展临空产业。完善临空产业规划，围绕航空物流、电子信息、健康医疗、智能制造等产业开展精准招商，重点引进临空特性高、产业带动性强的龙头企业落户。启动国际空港物流基地、临空产业园、燕矶航空小镇、汀祖航空物流产业园建设，为产业发展提供空间载体。学习借鉴国内航空港区和自贸区改革试点经验，营造法治化、国际化、便利化的营商环境。

　　推进配套基础设施和空港CBD建设。加快机场核心区水、电、路、气、通讯等基础设施的综合设计和建设。完善空港中央商务区（CBD）规划，全面启动体育中心、文化中心和会展中心等大型市政项目建设。启动112省道改扩建和花马湖水系改造。推动机场与周边高速公路、京九铁路、武黄城际铁路、长江黄金水道无缝衔接。

　　（二）深入推进供给侧结构性改革

　　持续推进“三去一降一补”。既去旧、又育新，有效减少无效供给，着力增加有效供给。用技术、安全、环保、市场等标准甄别落后产能和过剩产能，用市场化、法治化办法推进去产能。按照“保人不保企”的原则，坚决依法处置“僵尸企业”和闲置低效用地，为新经济释放发展空间。更多运用股权融资等直接融资方式，促进企业去杠杆。深化投融资体制改革，大力推广PPP模式，有效化解政府债务。严厉打击非法集资，解决居民杠杆率过高的问题。全面深化“放管服”，清理规范中介组织，落实国家减税降费政策，降低企业综合成本，提高市场主体获得感。投资重点向基础设施、技术改造、环境保护、实体经济、民生改善和公共服务等促进结构调整和转型升级的领域倾斜。加强对投资项目商业模式、融资模式、运营模式的设计和安排，确保投资项目可操作、能落地。

　　推动实体经济转型升级。实施百家企业上云计划，提升两化融合水平。运用技改扶持资金和产业基金支持传统工业升级改造，完成技改投资90亿元以上。实施中小企业成长计划，新增规上企业50家。新增“新三板”挂牌企业5家、“四板”挂牌企业20家。强力推进节能环保改造，实现钢铁行业在线监控，加快水泥行业整合。实施质量标准战略，推进质量强市和品牌强市建设。安排财政间歇资金10亿元，缓解实体企业融资难、融资贵的问题。

　　着力发展现代服务业。推动生产性服务业向专业化和价值链高端延伸，生活性服务业向精细和高品质转变。实施现代服务业成长工程。大力发展生产性服务业，做强做大葛店国家电子商务示范基地，培育临空物流产业，全面推进电商进农村。培育壮大金融业，力争金融业增加值达到14亿元。引进和支持国家节能建筑材料质量监督检验中心、湖北国信消防产品检测技术公司、长江检测认证公司等生产性服务业市场主体。加快发展高品质生活性服务业，科学修订商业网点规划，加快农贸市场、批发市场、休闲娱乐、住宿餐饮等改造升级。培植鄂州地方特色文化品牌，推进梧桐湖新区文化创意产业园建设。

　　推进农业供给侧结构性改革。大力发展生态农业，严格落实粮食安全行政首长负责制，加强高标准农田建设，确保粮食生产安全。加强农业品牌建设，狠抓农产品标准化生产、品牌创建和质量安全监管，做大做响“梁”字号系列有机农产品品牌，增加绿色优质农产品供给。围绕市场需求推进农业结构调整，着力优化产品结构、区域结构、经营结构，推广生态循环种养模式，大力发展名特优生态种业、高效经济林、花卉苗木等产业，加快发展农产品加工业。拓展农业多功能性，推进农业与旅游、教育、文化、健康养老等产业深度融合，发展观光农业、体验农业、创意农业等新业态。完善农业科技服务体系，实现科技对农业的贡献率超过62%。发展土地托管服务，推广农民“带地入股”，力争全市土地流转率达到40%以上。打造5个省级美丽宜居示范乡村。

　　（三）大力发展“四新经济”

　　构筑创新平台。推动市校创新合作常态化，加快推进华科大鄂州工研院、机器人与智能制造研究院、中国地大鄂州氢能产业园等创新平台建设。实施百家企业“走校进所”工程，加快科技型中小企业孵化器建设，支持企业建立研发机构，新增50个工业新产品，新增高新技术企业10家。突出绿色生态和科技创新主题，启动建设若干个具有产业特色、文化内涵、环境资源和服务功能的特色小镇。

　　集聚创新要素。充分发挥政府作为“天使投资人”作用，优化政府引导基金运行方式，针对项目特点，采取“补偿基金＋股权投资”、“引导基金＋担保机构”、“母基金＋社会投资”等多种方式促进新经济发展。鼓励商业银行对科技企业进行融资支持，大力推进投贷联动。强化人才第一支撑，建立“人才+项目+平台”的创新人才培养开发体系。依托东湖高新智力密集区，开辟高层次人才“绿色通道”，加快构建人才池。实施“333”高层次创新创业人才计划，着力引进一批能突破关键技术、能带动产业链形成的领军人才。持续实施“千名引硕工程”和“企业家培养工程”，完善创新创业平台和公共服务设施，最大限度消除地区间公共服务政策差距，为人才提供良好的创业环境和舒适的生活环境。

　　营造宽松环境。研究把握“四新经济”发展特点和趋势，减少对新经济发展的限制，放宽准入、降低门槛，大力营造鼓励创新、包容失败的社会氛围。抓住技术创新这个“牛鼻子”，突出应用导向和市场导向，用开放的眼光、灵活的政策、完善的服务扶持新经济发展，促进技术和传统产业融合，催生新模式、新业态。建立跨政府部门工作协调机制，整合技术、人才、资金和政策资源，全方位提升服务新经济发展的水平。

　　培育新兴产业。依据各开发区和新区特色，瞄准国内外前瞻领域、龙头企业、领军人才，主动对接招商，创造条件承载承接。葛店开发区、红莲湖新区、梧桐湖新区全面对接东湖高新区重大科技项目和特色园区，大力发展光电信息、微电子、生物医药、高端装备、人工智能、新能源等新兴产业。鄂州开发区、鄂城新区、花湖开发区重点谋划引进速度经济、即时经济和航空偏好型关联产业。主城区进一步优化城市服务功能，着力发展生产性服务业。

　　（四）加强生态建设和环境保护

　　实施生态修复工程。有序推进“生态修复、城市修补”工程。实施樊口二站、广家洲大堤加固、民信闸新建和洋澜湖生态补水等工程，启动五四湖南大堤，曹家湖、垱网湖、严家湖连通，红莲湖到大头海连通，青天湖水生态修复，三山湖大堤加固工程，推动樊口水利枢纽区域防洪及环境综合整治工程建设，筑牢水生态安全。实施矿山生态修复和地质灾害治理工程。加快推进生态公墓建设。加强长江沿岸滩涂复绿保护。大力推进天龙山公园一期、吴都生态廊道、莲花山文化主题公园、滨江风光带四期五期等生态园林工程。

　　加大环境治理力度。加强环保督察突出问题整改工作。加大环境保护执法力度，积极开展环保综合执法，综合治理扬尘、秸秆焚烧、机动车尾气等大气污染源。大力推广清洁能源，推进绿色公交全覆盖。系统推进水污染防治、水生态保护和水资源管理，全域禁止珍珠养殖、投肥养殖、围栏围网网箱养殖。大力开展畜禽养殖区域的环境整治，全面开展“一控两减三基本”行动，防治农业面源污染。大力开展黑臭水体治理，重点对余寿港、五丈港、新港河等进行专项整治。加快农村旱厕改造和污水管网建设，用两年时间实现集镇和村湾污水处理全覆盖。推进土壤污染防治工作。

　　完善绿色制度体系。以梁子湖全国生态文明试点和全国水生态文明建设试点为契机，建立城乡水资源统一管理和涉水事务一体化管理模式，建立全流域“河长制”。加强山体林地保护，建立更严格的保护制度。完善生态文明建设考核机制，探索绿色GDP动态考核。依据自然资源资产负债表，构建生态价值计量体系。综合运用领导干部自然资源资产离任审计结果。按照占补平衡原则，在全市范围内建立横向生态补偿制度。推进生态资产质押、收益权转让等融资方式创新，促进绿色金融产品创新和生态资产资本化。

　　（五）努力提升城市建设管理水平

　　坚持规划引领。推动城市规划由指导建设向规范建设、保护生态并重转变。推进“多规合一”国家级试点，全面划定生态红线、永久基本农田和城市开发边界。完成总规修编，实现控制性详规从主城区向新区全覆盖。强化规划实施和管理，坚持一张蓝图干到底，确保规划的权威性、严肃性和连续性。

　　提升城市功能和品质。推动城市建设由打造功能为主向提升功能、改善品质并重转变。实施“宽带中国示范城市”、“智慧鄂州”双推进计划，主城区重点区域实现“天网工程”全覆盖、重点公共场所实现WiFi全覆盖，城市、农村家庭宽带接入能力分别达到100兆和20兆。实施城市老旧小区环境提升工程，推动居民小区物业管理全覆盖。高标准推进海绵城市和地下综合管廊建设。加快重载车通道、凤凰大桥、东洋澜湖湿地公园二期、洋澜湖激光水秀、市民中心、图书馆、规划馆、档案馆、就业中心等重点工程建设。推进鄂咸高速、武阳高速鄂州段、武汉新港高速、鄂州机场高速建设。启动武汉地铁11号线延伸至葛店开发区工程，打通红莲湖与未来一路、未来三路瓶颈，做好科技一路对接葛店人民路和T9轨道延伸至梧桐湖前期研究。完成雨台山水厂、太和水厂工程，推进城东水厂建设。开工建设500千伏鄂州输变电工程、220千伏临江工程，在全省率先实现配电自动化系统全覆盖。

　　提高城市管理水平。优化城市管理组织体系，实行力量整合和管理重心下移。发挥社区在城市管理中的基础作用，提高社区自我管理和服务水平。推进环卫作业机械化，提升环卫保洁精细化水平。开展城市综合执法体制改革。编制城市管理标准化手册，推进城管标准化建设，实现城市管理网格化、执法规范化、作业精细化、考核常态化。深入推进“两车”综合整治，加强出租车、网约车运营管理和电动车规范管理。开展户外广告、渣土车等专项整治和治拥治堵专项行动。巩固旅游标准化示范城市和园林城市创建成果，实现国家卫生城市创建目标，积极推进全国文明城市、国家森林城市创建工作，建立美丽乡村建设长效机制。

　　（六）持续保障和改善民生

　　扎实推进脱贫攻坚。用率先全面小康建设带动精准扶贫。加大资源和政策整合力度，将扶贫政策不折不扣落到实处，切实增强贫困群众的获得感。努力探索产业扶贫的有效实现形式，探索“租金＋股金＋薪金”等模式，多渠道提高贫困群众收入水平，提高自我发展能力。加大扶贫督查考核验收和问责力度，确保年底１４个贫困村出列、10256名贫困人口销号。

　　加强就业和民生保障。实施更加积极的就业政策，推进高校毕业生、农村转移劳动力、零就业家庭等重点群体就业。整合创业扶持政策，实施创业引领计划，加大众创空间扶持力度。推进机关事业单位养老保险制度改革，完善五项保险制度，新增参保3万人次。推进社保一卡通工作。继续实施政府救助综合责任险，加强特困群众兜底保障工作，实现对困难群众社会救助全覆盖。加大货币化安置力度，新开工各类棚户区改造3.5万套，建成1.78万套。

　　发展各项社会事业。继续实施“全面改薄”工程，进一步改善办学条件。新建、改扩建12所普惠性幼儿园。优化主城区学校规划布局，有序化解大班额问题。启动城东学校建设，加快恒大学校建设，完成市二中、鄂州中专迁建一期工程。支持鄂州职业大学创建应用型本科院校。启动电商大学、华师大梁子湖校区建设。加快精准医疗中心工程建设，建成湖北中医院武东院区，启动市儿童医院建设。探索组建市中心医院医疗集团。实施基本生育免费服务。依法实施全面两孩政策，优化出生人口性别比。扎实开展文化惠民、全民健身、全民阅读等公共文化体育活动。完善现代公共文化服务体系，推进基本公共体育服务体系示范区建设。深度挖掘鄂州地方特色文化，创作一批艺术精品。

　　创新社会治理。完善城市社区和村民委员会治理体系，加强基层组织建设，提升基层社会治理水平。大力培育社会组织，加强社会组织管理和社会工作人才队伍建设。构建全市统一的流动人口综合信息平台。建立安全生产“双重预防性”工作机制，加强社会治安防控体系建设，大力提升群众“一感两度两率”。建立完善守信联合激励和失信联合惩戒制度，加快社会诚信建设。完善食药监管体系改革，逐步建立食品药品市场准入和追溯体系。强化市场监管，维护公平有序的交易环境和安全放心的消费环境。加快推进公路安全生命防护工程，强化应急管理，提高防灾减灾、处置突发事件能力。加强风险研判，强化源头治理，完善“1+N”社会矛盾纠纷预防化解机制，不断提升信访维稳工作水平，确保社会大局和谐稳定。

　　（七）加快推进重点领域改革

　　围绕治理能力和治理体系推进改革。探索建立有利于统筹城市空间管理、市场管理、民生社会、水务管理、综合执法的管理体制，推动城市治理体系和治理能力现代化。搭建PPP管理架构，规范管理流程，在基础设施建设、公共服务、资源环境、生态建设等领域推进社会资本与政府开展合作。深入推进国有资产归集、运营、处置制度改革。改革工业用地配置制度，探索并逐步将土地一次性出让改为分期分阶段出让、先租后让、年租等弹性供地方式。

　　深化农村改革。分类实施农村集体资产确权到户和股份制改革，深入推进农村土地“三权分置”，引导多种形式适度规模经营，培育新型农业经营主体和服务主体。完善农业发展投融资平台和农村综合产权交易平台，推动农村产权交易和抵押融资。深化供销合作社综合改革。完成国有林场改革任务。有序推进被征地农民市民化改革。

　　开展改革“回头看”。对市级权力下放、公共服务下沉、电子政务在线审批、公共信息共享等问题比较集中、阻力比较大的改革试点，进行考核评估，研究解决制约改革的深层次体制机制问题，进一步优化试点方案，加大推进力度，坚定不移减少政府对微观事务管理，切实提高服务水平。

　　对改革经验进行总结推广。系统总结统筹城乡发展、农村产权制度改革、公共服务下沉、城乡网格化管理、“放管服”等领域改革的经验，针对试点过程中出现的问题，健全制度、优化流程、完善方式，促进在更大范围内复制推广。

　　四、切实加强政府自身建设

　　各位代表，全面完成市第七次党代会确定的各项目标任务，扎实推进国际航空大都市建设，政府肩负着重大责任。我们一定切实加强自身建设，不断提高政府治理现代化水平，努力建设让人民满意的政府。

　　（一）全面推进依法行政。加强政府法治工作，健全依法决策机制，推行重大行政决策合法性审查制度，全面开展规范性文件清理，做好地方立法工作。深入推进法律顾问制度，探索成立鄂州市仲裁委员会。进一步推进行政复议委员会工作。加大政务公开力度，构建适应新媒体的政府信息公开互动机制。认真落实市人大及其常委会各项决定、决议，办理好人大代表建议和政协提案工作，自觉接受人大工作监督、法律监督和政协民主监督，自觉接受社会公众和舆论监督。

　　（二）着力提升服务效能。加快电子政务建设，打造政务数据资源共享平台，继续推进线上线下政务服务向基层延伸，最大程度利民便民。进一步树立政府公务人员的敬业服务精神，提升素质能力。优化行政决策程序和服务流程，提高各级政府决策服务效能。进一步精简会议和文件，办有效的事，开务实的会，发管用的文。严格绩效管理，健全与五大发展理念相一致的目标考核评价奖惩体系。

　　（三）切实转变工作作风。加强对重大决策部署、重大改革措施执行情况的监督检查，确保令行禁止。深入贯彻中央八项规定精神，持续加大“四风”整治力度，强化领导干部经济责任审计结果运用，推进作风建设常态化、长效化。强化责任担当，加大问责力度，坚决整治庸政、懒政、怠政。健全容错纠错机制，强化正向引导，激励干事创业。

　　（四）推进廉洁政府建设。认真贯彻落实《关于新形势下党内政治生活的若干准则》和《中国共产党党内监督条例》等党内法规，推动政府系统“两个责任”落地生根，实现有权必有责、有责要担当、失责必追究。深入推进源头防腐，加强党风廉洁教育，强化权力运行监督制约，完善廉洁风险防控机制，做到用制度管权管事管人。持续保持惩治腐败的高压态势，依纪依法严肃查处违法乱纪行为。

　　各位代表，建设国际航空大都市使命光荣、责任重大、任务艰巨，时不我待。全市人民的信任和期盼，鞭策我们义无反顾、勇往直前。我们要更加紧密地团结在以习近平同志为核心的党中央周围，在省委、省政府和市委的坚强领导下，全力以赴推动市委重大战略部署项目化、方案化、具体化，忘我拼搏，只争朝夕，以新的精神状态、新的工作举措、新的业绩作为，向全市人民交上一份新的满意答卷！
